# Early hypoxia-induced secretome remodeling reveals adaptive mechanisms and biomarkers of blood-brain barrier dysfunction in ischemic stroke

**DOI:** 10.1186/s13041-026-01283-5

**Published:** 2026-02-16

**Authors:** Qian Wu, Yao Jiang, Lingling Peng, Yingqiang Dang, Chongge You, Xiaoxin Li

**Affiliations:** Laboratory Medicine Center, The Second Hospital & Clinical Medical School, Lanzhou University, Lanzhou, China

**Keywords:** Blood-brain barrier dysfunction, Ischemic stroke, Brain microvascular endothelial cell, Adaptive response, Secretome, Hypoxic stress, Circulating biomarkers

## Abstract

Reversible disruption of the blood-brain barrier (BBB) occurs within hours after the onset of ischemic stroke (IS), offering a critical window for therapeutic intervention. However, the molecular characteristics and their potential as circulating biomarkers associated with this transient phase of BBB dysfunction remain poorly defined. To elucidate these mechanisms, we employed an oxygen-glucose deprivation (OGD) model in human cerebral microvascular endothelial cells (hCMEC/D3) to simulate early ischemic stress, and systematically profiled their secreted proteome and metabolome. By comparing with non-brain-derived human umbilical vein endothelial cells (HUVECs), we identified brain endothelium-specific hypoxic response signatures. These molecules were significantly enriched in pathways related to metabolic reprogramming, antioxidant defense, and epigenetic regulation pathways, indicating a coordinated adaptive response to preserve BBB homeostasis. Furthermore, integrative multi-omics analysis revealed 14 protein-metabolite pairs with potential functional synergy. Based on a multi-criteria screening strategy including brain specificity, functional relevance, and secretory potential, we prioritized 10 candidate circulating biomarkers: ALDH2, ITGA5, KYNU, TFRC, CD44, COL1A2, HEXB, HSPG2, THBS4, and DLD. Preliminary validation using serum from acute IS (AIS) patients and healthy controls showed significantly altered levels of ALDH2, ITGA5, KYNU, and TFRC, with TFRC exhibiting promising diagnostic performance both individually (AUC = 0.816) and in combination with the other three biomarkers (AUC = 0.876). Moreover, multivariate logistic regression analysis revealed that elevated TFRC was independently associated with poor 90-day outcomes (OR = 1.02, 95% CI 1.00–1.04, *P* = 0.031), while higher DLD levels were correlated with good prognosis (OR = 0.68, 95% CI 0.39–0.90, *P* = 0.047). Notably, TFRC expression was upregulated in hCMEC/D3 cells under early hypoxic stress, while its extracellular secretion was reduced. This observation suggests a potential early cellular adaptation to preserve iron homeostasis. In summary, these findings uncover early molecular adaptations of brain microvascular endothelial cells to ischemic stress and propose a panel of secreted biomarkers with translational potential for early diagnosis and outcome prediction in IS, potentially guiding the development of time-sensitive therapeutic strategies.

**Supplementary Information:**

The online version contains supplementary material available at 10.1186/s13041-026-01283-5.

## Introduction

Ischemic stroke (IS) is one of the leading causes of disability and death worldwide, and its pathogenesis involves an imbalance of multiple components within the neurovascular unit (NVU) [1–3]. The blood brain barrier (BBB), a core component of the NVU, plays a crucial role in the progression and repair of stroke-related brain injury by maintaining its structural and functional integrity [4, 5]. Studies have shown that BBB permeability is altered within the first 3 h of IS onset [6]. The destructive process can be broadly divided into two stages: early reversible dysfunction (within hours after ischemia) and late irreversible structural damage (24–72 h post-ischemia) [7, 8]. Sustained BBB disruption not only exacerbates the local inflammatory cascade response, inducing cerebral edema and neuronal injury, but also correlates with post-stroke cognitive impairment and increased risk of recurrence [9–11]. Therefore, identifying biomarkers of the reversible BBB dysfunction may support timely clinical decision-making and personalized interventions during the acute phase of IS.

Human brain microvascular endothelial cells (HBMECs), which form the structural and functional core of the BBB, preserve central nervous system (CNS) homeostasis through tight junctions and selective molecular transport [12]. Although primary HBMECs-based hypoxia models are valuable for studying early BBB responses to ischemic stress, their limited proliferative capacity and technical challenges in culture restrict their application. The immortalized human brain microvascular endothelial cell line hCMEC/D3 has been widely used as an in vitro model of the BBB due to its well-defined origin, ease of culture, and stable phenotype [13]. In contrast, human umbilical vein endothelial cells (HUVECs), lacking the specialized BBB properties, serve as a representative non-brain origin endothelial cell type [14]. Therefore, in this study, hCMEC/D3 cells were used to model BBB responses to hypoxic stress, while HUVECs were employed as a non-brain endothelial control to identify BBB endothelial cell-specific patterns of hypoxia-responses and potential biomarkers.

Under ischemic conditions, proteins and metabolites secreted by HBMECs play key roles in mediating intercellular communication and reflecting endothelial pathophysiological states [15]. Following BBB disruption, these secreted molecules can enter the peripheral circulation, highlighting their potential as serum biomarkers for hypoxic BBB injury. Previous studies have applied techniques such as stable isotope labeling with amino acids in cell culture (SILAC) and data-dependent acquisition mass spectrometry (DDA-MS) to profile the secretome of hCMEC/D3 cells under OGD conditions, identifying 727 [16] and 1200 [17] secreted proteins, respectively. Recent advances in high-resolution proteomics platforms, particularly four-dimensional data-independent acquisition (4D-DIA) MS, have significantly improved both the depth and throughput of protein detection [18], providing enhanced capabilities for biomarker discovery. However, compared to the proteome, the metabolic signatures of hCMEC/D3 cell supernatants under oxygen-glucose deprivation (OGD) conditions remain poorly characterized. This under-investigated area restricts our insights into hypoxia-induced metabolic reprogramming and novel metabolite-based biomarker discovery.

In addition, although single-omics analyses can identify a large number of candidate molecules, it is difficult to capture complex functional interactions between biomolecules, which limits the efficiency and interpretability of biomarker discovery. Integrated proteomic and metabolomic analyses, by constructing protein-pathway-metabolite networks, enable the identification of biologically coordinated molecular pairs. This strategy may offer a more comprehensive framework for identifying functionally relevant biomarkers [19, 20].

To fill the gap in understanding early secretome alterations of HBMECs under ischemic stress, and explore the specific circulating biomarkers that reflect early and potentially reversible BBB dysfunction. We performed integrated 4D-DIA proteomics and untargeted metabolomics profiling of hCMEC/D3 cell supernatants following short-term OGD. Using HUVECs as non-brain endothelial controls, we aimed to distinguish brain endothelium-specific hypoxic response signatures. Furthermore, integrative proteomic-metabolomic analysis combined with a multi-criteria biomarker screening strategy was used to prioritize candidate molecules of early BBB dysfunction. Selected candidate biomarkers were subsequently validated for their clinical relevance in acute IS (AIS) patient serum within 24 h of onset and healthy controls. This study seeks to provide novel insights into early BBB hypoxic responses and uncover potential circulating biomarkers for early diagnosis and functional outcome prediction in IS.

## Methods

### Cell culture and OGD modeling

hCMEC/D3 cells and HUVECs were obtained from the Pathology Research Institute of West China Hospital, Sichuan University. Cells were cultured in Endothelial Cell Medium (ScienCell, USA) supplemented with endothelial cell growth supplement (ECGS) and 5% fetal bovine serum (FBS). Cultures were maintained at 37 °C in a humidified incubator with 5% CO₂. Before OGD treatment, hCMEC/D3 cells and HUVECs were washed three times with phosphate-buffered saline (PBS), then resuspended in glucose- and serum-free Dulbecco’s Modified Eagle Medium (DMEM). Cells were incubated for 6 h in a hypoxia chamber (Series II Water Jacket, Thermo Scientific, USA) under a gas mixture of 1% O₂, 5% CO₂, and 94% N₂. Control groups were maintained under normoxic conditions and served as references.

### Cell supernatant collection and drying

The cell supernatants were collected into 50 mL centrifuge tubes and centrifuged sequentially to remove cells, cell debris, and extracellular vesicles: 300 g at 4 °C for 10 min; 2000 g at 4 °C for 10 min; 10,000 g at 4 °C for 30 min; and 100,000 g at 4 °C for 70 min. The clarified supernatants were concentrated and dried using a vacuum freeze dryer. The lyophilized products were stored in sterile centrifuge tubes until further analysis.

### 4D-DIA quantitative proteomic analysis

Samples were ground in liquid nitrogen and subjected to phenol extraction using a Tris-buffered phenol solution containing protease and phosphatase inhibitors. Proteins were precipitated with ammonium acetate in methanol, washed with methanol and acetone, and redissolved in lysis buffer. After quantification, equal amounts of proteins were reduced with dithiothreitol (DTT), alkylated with iodoacetamide, acetone-precipitated, and digested with trypsin overnight at 37 °C. Peptides were desalted using SOLA™ SPE plates and spiked with iRT standards (Biognosys, Thermo Fisher). Liquid chromatography-tandem mass spectrometry (LC-MS/MS) analysis was performed on a timsTOF Pro mass spectrometer (Bruker, Germany) coupled with an UltiMate 3000 RSLCnano system (Thermo Scientific, USA). Peptides were separated on a 15 cm × 75 μm C18 column at 300 nL/min. The linear gradient was set as follows: 0–48 min, 5–22% B; 48–53 min, 22–35% B; 53 ~ 56 min, 35–90% B;56–57 min, 90 − 3% B; 57–60 min, 3% B. Ion mobility is set from 0.7 to 1.3 V·s/cm2 and the collision energy range from 20 to 59 eV. MS/MS spectra acquired from 100 to 1700 m/z. Raw mass spectrometry data were analyzed using Spectronaut Pulsar (version 18.4, Biognosys, Switzerland).

### Non-targeted metabolomics analysis

Lyophilized samples (10 mg) were homogenized in 400 µL methanol/water (4:1, v/v, with 4 µg/mL internal standards) using steel beads. After − 40 °C pre-cooling and grinding, samples were sonicated in ice water for 10 min and incubated overnight at − 40 °C. Supernatants were collected after centrifugation (12,000 rpm, 20 min, 4 °C), and 150 µL was used for LC-MS analysis. Quality control (QC) samples were prepared by pooling equal aliquots from all samples. LC-MS analysis was performed on a Waters ACQUITY UPLC I-Class Plus system (Waters, USA) coupled with a Thermo Q Exactive HF mass spectrometer (Thermo, USA) in both positive and negative ESI modes. Metabolites were separated on an ACQUITY UPLC HSS T3 column (100 mm × 2.1 mm, 1.8 μm) using a binary gradient at a 0.35 mL/min flow rate. QC samples were injected periodically to monitor system stability. Raw data were processed with Progenesis QI v3.0 (Nonlinear Dynamics, UK), including peak picking, alignment, retention time correction, and normalization. Metabolites were identified by matching to The Human Metabolome Database (HMDB, https://hmdb.ca/), Lipidmaps (https://lipidmaps.org/), METLIN (https://metlin.scripps.edu/), and our local database.

### Participants and sample collection

A total of 548 patients with AIS admitted to the the Second Hospital & Clinical Medical School, Lanzhou University between January 2023 and February 2024, and 254 healthy controls were enrolled. From these participants, 65 AIS patients (AIS group) and 65 age- and gender-matched controls (control group) were randomly selected for analysis. The inclusion and exclusion criteria for patients have been described in detail in our previously published study [21]. This study was performed in accordance with the 2013 Declaration of Helsinki and was approved by the Ethics Committee of the Second Hospital of Lanzhou University (IRB numbers: 2023 A-034 and 2024 A-331). All participants ensured their privacy by signing an informed consent form.

Blood was collected and tested immediately after the admission of patients with suspected AIS. In addition, blood samples were collected from healthy controls after fasting for more than 8 h. The collected peripheral blood samples were centrifuged (3500 g, 15 min, 4 °C) to separate the serum and stored at − 80 °C until analysis. Clinical information and National Institutes of Health Stroke Scale (NIHSS) scores of the AIS patients at admission were collected from electronic medical records. Functional outcomes were assessed at 90 days post-stroke using the modified Rankin Scale (mRS) via telephone follow-up. The AIS group was categorized into a good prognosis subgroup (mRS ≤ 2) and a poor prognosis subgroup (mRS > 2) [22, 23].

### Serum biomarker quantification

The expression levels of 10 candidate biomarkers (ALDH2, ITGA5, KYNU, TFRC, CD44, COL1A2, HEXB, HSPG2, THBS4, DLD) in the serum were measured using enzyme-linked immunosorbent assay (ELISA) kits (FineTest, China). The assay was repeated twice for each sample, and the mean value was used for statistical analysis. Intra-assay and inter-assay coefficients of variation (CV) for all assays were below 20%, confirming good reproducibility and reliability.

### Immunofluorescence and confocal microscopy

After normoxic or OGD treatment, hCMEC/D3 cells cultured on glass-bottom dishes were fixed with 4% paraformaldehyde and blocked with 10% goat serum. Cells were then incubated with primary antibody against TFRC (HUA BIO, Hangzhou, China), followed by fluorescein isothiocyanate (FITC)-conjugated secondary antibody (Proteintech, Wuhan, China). Nuclei were counterstained with 4’,6-diamidino-2-phenylindole (DAPI). Images were acquired using a Zeiss LSM880 laser scanning confocal microscope (Carl Zeiss, Germany) equipped with 20× and 63× oil immersion objectives. Identical acquisition settings were applied to all groups. Quantification of TFRC fluorescence intensity was performed using ZEN Blue software (Carl Zeiss, Germany).

### Western blotting of cell lysates and supernatants

For analysis of intracellular TFRC expression, hCMEC/D3 cells were lysed using radio-immunoprecipitation assay (RIPA) buffer supplemented with protease inhibitors. Protein concentrations were quantified using a bicinchoninic acid (BCA) assay, and equal amounts of total protein were subjected to sodium dodecyl sulfate-polyacrylamide gel electrophoresis (SDS-PAGE) and transferred to polyvinylidene fluoride (PVDF) membranes. Then the membranes were incubated with primary antibodies against TFRC (HUA BIO, Hangzhou, China) and β-actin (Abcam, Cambridge, MA, USA), followed by horseradish peroxidase (HRP)-conjugated secondary antibody (Proteintech, Wuhan, China). Bands were visualized using enhanced chemiluminescence (ECL) and imaged with an ECL detection system.

To assess secreted TFRC, culture supernatants were collected and concentrated using a vacuum freeze-dryer. Equal volumes of buffer were separated by SDS-PAGE. One gel was stained with Coomassie Brilliant Blue to evaluate total protein content, while the other was used for Western blotting to detect TFRC. Band intensity was normalized to total protein levels. Densitometric analysis of band intensities was performed using ImageJ software (Rawak Software Inc., Stuttgart, Germany).

### Statistical analysis

Proteomic data were assessed for differential expression using Student’s t-test, followed by Benjamini–Hochberg correction to obtain false discovery rates (FDR). Differentially expressed proteins (DEPs) were defined as those with |log_2_ fold change (log_2_FC)| ≥ 1 and *P* < 0.05. Metabolomic data were analyzed using multivariate statistical approaches. Principal component analysis (PCA) was used to evaluate sample distribution and system stability. Orthogonal partial least squares-discriminant analysis (OPLS-DA) was applied to distinguish group differences, and model robustness was assessed via 7-fold cross-validation and 200-permutation tests. Variable importance in projection (VIP) value was used to rank the overall contribution of each metabolite to group differentiation. Differentially expressed metabolites (DEMs) were identified based on the criteria of VIP > 1.0, *P* < 0.05, and |log_2_FC| ≥ 1. Gene Ontology(GO)and Kyoto Encyclopedia of Genes and Genomes༈KEGG༉enrichment analyses were conducted for both DEPs and DEMs using the hypergeometric test, with FDR correction applied to determine significantly enriched pathways (*q* < 0.05). Shared KEGG pathways between DEPs and DEMs were visualized via Venn diagrams. Protein-pathway-metabolite networks were constructed to identify functional associations, and protein-metabolite pairs were further extracted based on KEGG pathway maps.

For clinical data, normally distributed continuous variables were expressed as mean ± SD and compared using Student’s t-test, while non-normal variables were reported as median (IQR) and compared using the Mann-Whitney U test. Categorical variables were presented as frequencies (n, %) and analyzed using chi-square or Fisher’s exact test. Correlations were assessed using the Spearman test. The prognostic value of candidate biomarkers was evaluated via univariate and multivariate logistic regression models. Receiver operating characteristic (ROC) curves and area under the curve (AUC) analyses were used to assess diagnostic and prognostic performance. All statistical analyses were conducted in R (Version 4.4.3), with a two-tailed *P* < 0.05 being considered statistically significant.

## Results

### Proteomic analysis

#### Proteomic profiling of hCMEC/D3 and HUVEC under OGD conditions

**Fig. 1:**
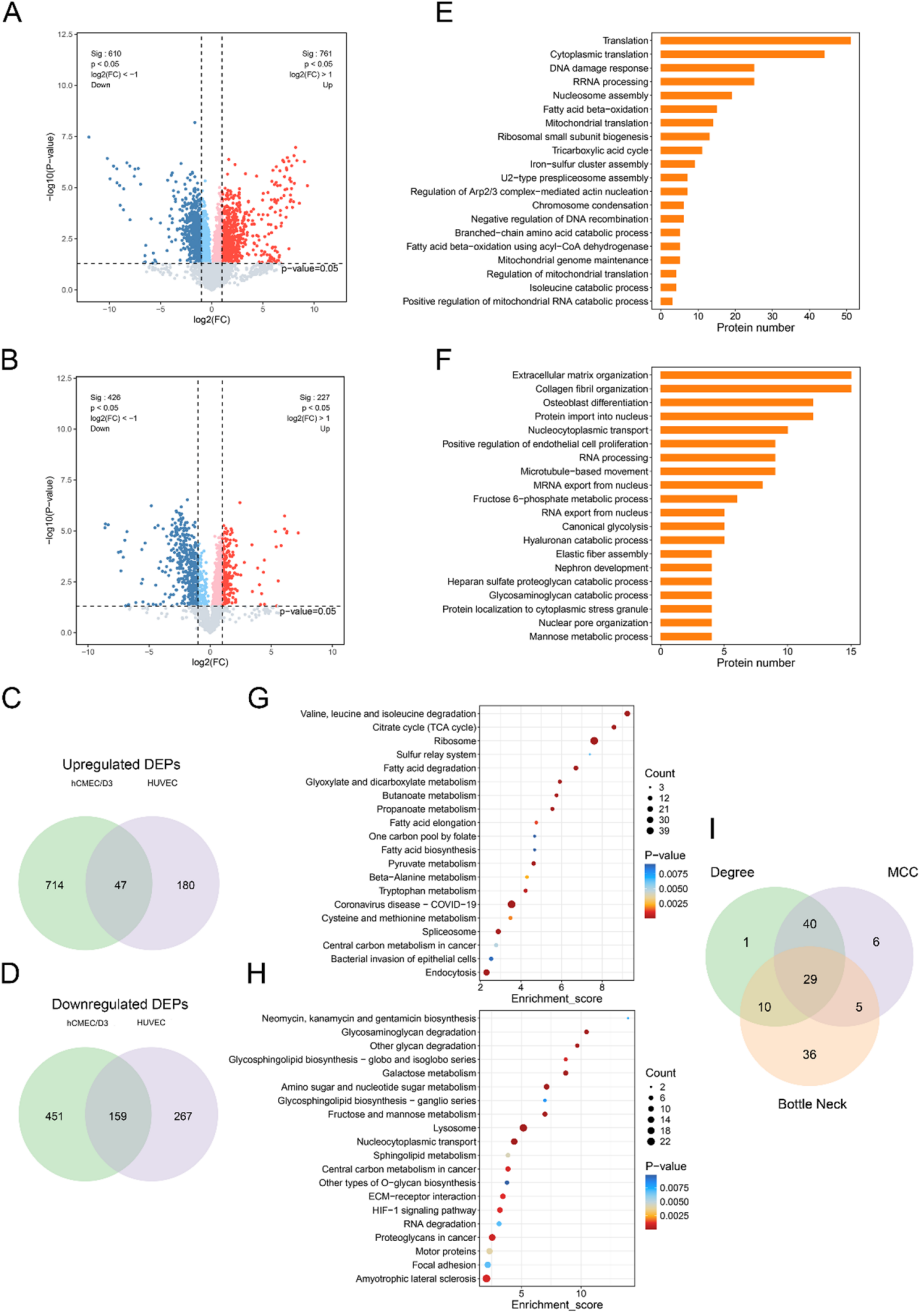
Proteomic profiling of OGD-induced secretomes from hCMEC/D3 cells and HUVECs reveals brain endothelium-specific hypoxic responses. **A**, **B** Volcano plots showing differentially expressed secreted proteins in hCMEC/D3 cells (**A**) and HUVECs (**B**) following oxygen-glucose deprivation (OGD) compared to normoxic control. Red and blue dots represent significantly upregulated and downregulated proteins, respectively. Gray dots represent non-significant proteins (*P* > 0.05). **C**, **D** Venn diagrams illustrate the intersection analysis of differentially expressed proteins (DEPs) between hCMEC/D3 cells and HUVECs. **C** upregulated DEPs and **D** downregulated DEPs. **E**–**H** Biological function and pathway enrichment of hCMEC/D3-specific secretory proteins under OGD. **E** Gene Ontology Biological Process (GOBP) for upregulated DEPs; **F** GOBP for downregulated DEPs; **G** Kyoto Encyclopedia of Genes and Genomes (KEGG) pathway analysis for upregulated DEPs. **H** KEGG pathway analysis for downregulated DEPs. **I** Venn diagram showing the overlap of key proteins identified by three algorithms (Degree, Maximal Clique Centrality, Bottleneck) in the protein-protein interaction network analysis

Compared to the controls, a total of 1371 DEPs were screened in the supernatant of hCMEC/D3 cells following OGD treatment, including 761 up-regulated and 610 down-regulated proteins (Fig. 1A). In HUVECs, 653 DEPs (227 upregulated, 426 downregulated) were detected under the same conditions (Fig. 1B). To identify proteins specifically secreted in hCMEC/D3 cells in response to OGD, we further performed intersection analyses of DEPs between hCMEC/D3 cells and HUVECs. The results showed that there were 714 up-regulated DEPs and 451 down-regulated DEPs in the secretome of hCMEC/D3 cells compared to the HUVECs (Fig. 1C, D), reflecting a distinct hypoxia-induced secretory response of brain microvascular endothelial cells.

#### Functional enrichment analysis

To explore the potential functions of hCMEC/D3-specific secreted proteins under hypoxic stress, Gene Ontology biological process (GOBP) enrichment analysis was first performed. The results revealed that 714 up-regulated DEPs were mainly enriched in a variety of biological processes related to protein synthesis. Such as cytoplasmic translation, translation, nucleosome assembly, and other processes. Meanwhile, these up-regulated proteins were also significantly involved in energy metabolism processes, such as fatty acid beta-oxidation, and tricarboxylic acid cycle (TCA cycle). The 451 down-regulated DEPs were involved in glycolytic processes (fructose 1,6-bisphosphate metabolic process, mannose metabolic process) and extracellular matrix (ECM) related processes (collagen fibril organization, extracellular matrix organization). Additionally, nuclear transport and stress granule assembly processes (protein import into the nucleus, nucleocytoplasmic transport, protein localization to cytoplasmic stress granule) were also significantly enriched in down-regulated DEPs (Fig. 1E, F).

Consistently, KEGG pathway enrichment analysis further confirmed the key biological processes highlighted by the GOBP results. The up-regulated DEPs were significantly enriched in pathways related to protein synthesis and metabolism regulatory pathways, including the ribosome pathway, amino acid metabolism (valine, leucine, and isoleucine degradation, tryptophan metabolism, and cysteine and methionine metabolism), carbohydrate metabolism (TCA cycle, pyruvate metabolism), and fatty acid metabolism (fatty acid degradation and fatty acid elongation). The down-regulated DEPs were significantly enriched in the lysosome pathway and several carbohydrate metabolic pathways, such as amino sugar and nucleotide sugar metabolism, galactose metabolism, and fructose and mannose metabolism. Additionally, ECM-receptor interaction and hypoxia-inducible factor-1 (HIF-1) signaling pathways were also significantly enriched (Fig. 1G, H).

#### Identification of hub hypoxic response proteins in hCMEC/D3

To identify hub hypoxia-induced proteins in hCMEC/D3 cells, we screened 28 significant pathways with *q*-value < 0.05 based on the results of previous KEGG enrichment analysis (Table S1). Subsequently, all DEPs enriched in these pathways were extracted, and duplicates were removed, resulting in 222 proteins. Next, the protein-protein interaction (PPI) networks of these proteins were constructed using the STRING database (https://string-db.org/) (Fig. S1). The interaction data were imported into Cytoscape software (v3.10.3) for network topology analysis. Three algorithms, Degree, Maximum Clique Centrality (MCC), and Bottleneck, were used to identify potential hub proteins through the CytoHubba plug-in. Among the top 80 ranked proteins from each algorithm, a total of 29 proteins were consistently identified by all three algorithms (Fig. 1I), with detailed information summarized in Table 1.

**Table 1:** Characteristics of 29 hub proteins involved in the hypoxic response of hCMEC/D3

Symbol	Protein name	log_2_FC	Regulation	q-value
CS	Citrate synthase, mitochondrial	3.300	Up	3.62E−04
SDHA	Succinate dehydrogenase [ubiquinone] flavoprotein subunit A, mitochondrial	1.560	Up	4.20E−02
ACO2	Aconitate hydratase, mitochondrial	2.682	Up	1.12E−03
SUCLA2	Succinate-CoA ligase [ADP-forming] subunit beta, mitochondrial	2.715	Up	4.58E−02
IDH2	Isocitrate dehydrogenase [NADP], mitochondrial	2.137	Up	3.63E−02
DLAT	Dihydrolipoyllysine-residue acetyltransferase component of pyruvate dehydrogenase complex, mitochondrial	5.847	Up	9.59E−02
SNRPD2	Small nuclear ribonucleoprotein Sm D2	1.305	Up	1.93E−02
DLD	Dihydrolipoyl dehydrogenase, mitochondrial	1.358	Up	6.61E−03
MDH2	Malate dehydrogenase, mitochondrial	1.859	Up	1.09E−03
ALDH2	Aldehyde dehydrogenase, mitochondrial	1.583	Up	2.21E−02
GOT2	Aspartate aminotransferase, mitochondrial	1.655	Up	4.36E−03
CD44	CD44 antigen	−1.120	Down	4.46E−03
ITGA5	Integrin alpha-5	−1.339	Down	3.56E−02
PFKM	ATP-dependent 6-phosphofructokinase, muscle type	−1.695	Down	4.25E−03
BCAT2	Branched-chain-amino-acid aminotransferase, mitochondrial	3.932	Up	6.74E−03
KYNU	Kynureninase	1.249	Up	6.74E−03
TFRC	Transferrin receptor protein 1	−1.525	Down	4.08E−03
LUM	Lumican	−2.476	Down	7.74E−03
COL1A2	Collagen alpha-2(I) chain	−1.536	Down	2.27E−02
ACADM	Medium-chain specific acyl-CoA dehydrogenase, mitochondrial	1.759	Up	5.74E−02
HEXB	Beta-hexosaminidase subunit beta	−1.769	Down	1.33E−03
HK1	Hexokinase-1	−3.273	Down	1.09E−03
HSPG2	Basement membrane-specific heparan sulfate proteoglycan core protein	−1.517	Down	3.71E−03
MCCC1	Methylcrotonoyl-CoA carboxylase subunit alpha, mitochondrial	6.130	Up	6.83E−04
SHMT2	Serine hydroxymethyltransferase 2, mitochondrial	1.698	Up	1.59E−03
THBS4	Thrombospondin-4	−1.047	Down	3.75E−02
HIBADH	3-hydroxyisobutyrate dehydrogenase, mitochondrial	2.445	Up	4.31E−03
LSM3	U6 snRNA-associated Sm-like protein	1.239	Up	3.07E−02
SEH1L	Nucleoporin SEH1	−1.539	Down	2.38E−03

### Metabolomic analysis

#### Metabolic profiling of hCMEC/D3 and HUVEC under OGD conditions

**Fig. 2:**
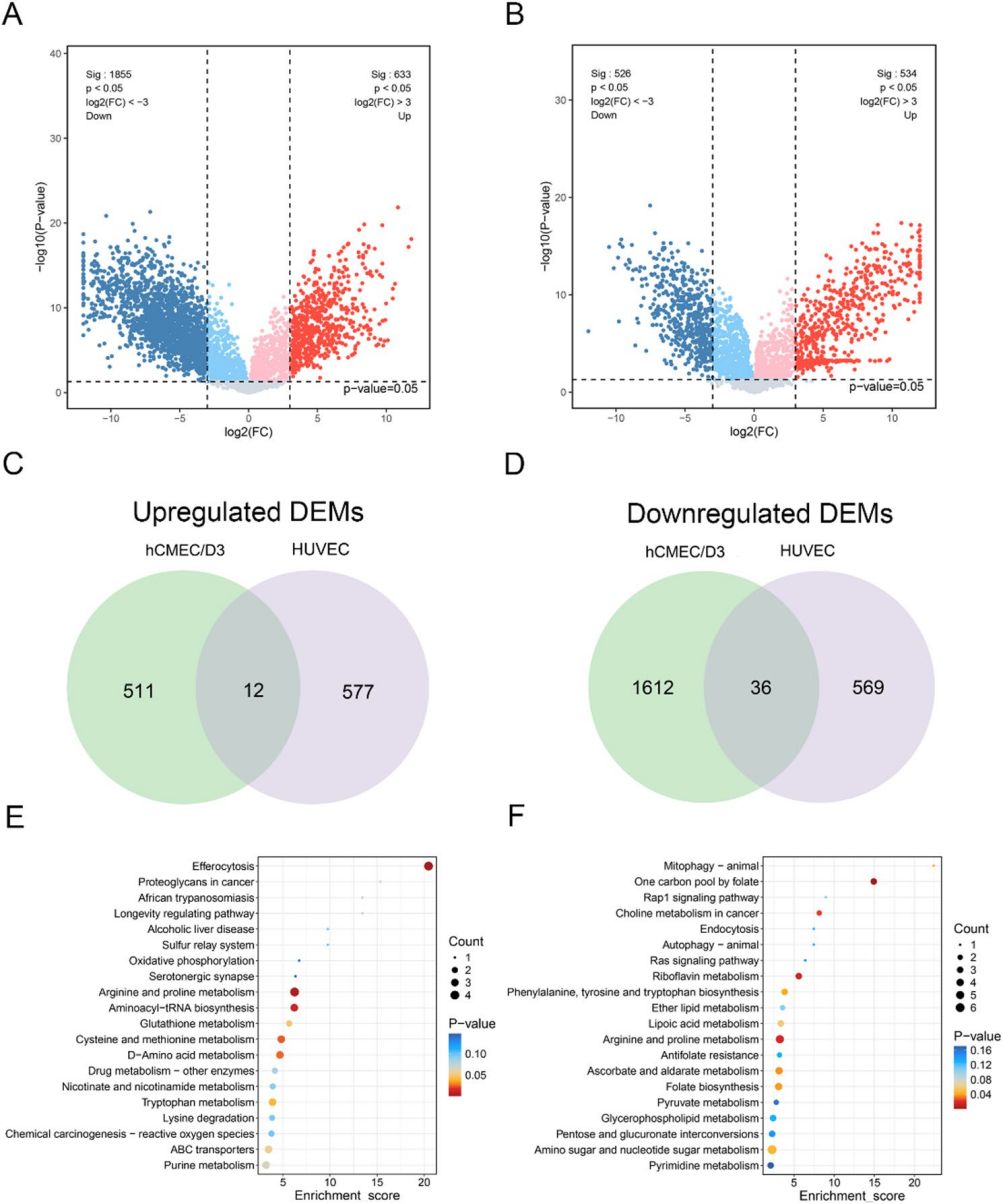
Metabolomic profiling of OGD-treated hCMEC/D3 cells and HUVECs. **A**, **B** Volcano plots showing differentially expressed metabolites (DEMs) in the conditioned media of hCMEC/D3 cells (**A**) and HUVECs (**B**) following oxygen-glucose deprivation (OGD). **C**, **D** Venn diagrams illustrate the overlap of upregulated and downregulated DEMs between hCMEC/D3 cells and HUVECs. **E**, **F** Kyoto Encyclopedia of Genes and Genomes (KEGG)pathway enrichment analysis of hCMEC/D3-specific DEMs in response to OGD. **E** KEGG pathway analysis for upregulated DEMs; **F** KEGG pathway analysis for downregulated DEMs

PCA and OPLS-DA analysis demonstrated a clear separation trend between the OGD-treated and control groups in both hCMEC/D3 cells and HUVECs (Fig. S2A-D). To evaluate the quality of the OPLS-DA models, 200 permutation tests were performed, and the results indicated that both models had good performance without overfitting (Fig. S2E-F). A total of 2171 DEMs were identified in the supernatants of hCMEC/D3 cells following OGD treatment based on the criteria of VIP ≥ 1, |log₂FC| ≥ 1, and *P* < 0.05. Among these, 523 were up-regulated metabolites and 1648 were down-regulated metabolites (Fig. 2A). In contrast, 1194 DEMs were identified in HUVECs, with 589 up-regulated and 605 down-regulated (Fig. 2B). Subsequent intersection analysis of the DEMs between the two cell types indicated that, under OGD conditions, hCMEC/D3 cells exhibited a distinct extracellular metabolic profile, characterized by 511 up-regulated and 1612 down-regulated metabolites relative to HUVECs (Fig. 2C, D).

#### Functional enrichment analysis

To further elucidate the metabolic response of hCMEC/D3 cells to hypoxic stress, KEGG pathway enrichment analysis was performed on the cell-specific DEMs. The results revealed that the 511 up-regulated DEMs were most significantly enriched in the Efferocytosis pathway. In addition, several amino acid metabolism-related pathways, including Arginine and proline metabolism, Tryptophan metabolism, and Cysteine and methionine metabolism, were also significantly enriched. Enrichment was also observed in nucleotide metabolism pathways such as Purine metabolism and Pyrimidine metabolism (Fig. 2E). The 1,648 down-regulated DEMs were predominantly enriched in folate metabolism-related pathways, including One carbon pool by folate and Folate biosynthesis (Fig. 2F).

### Integrative analysis of proteome and metabolome

**Fig. 3:**
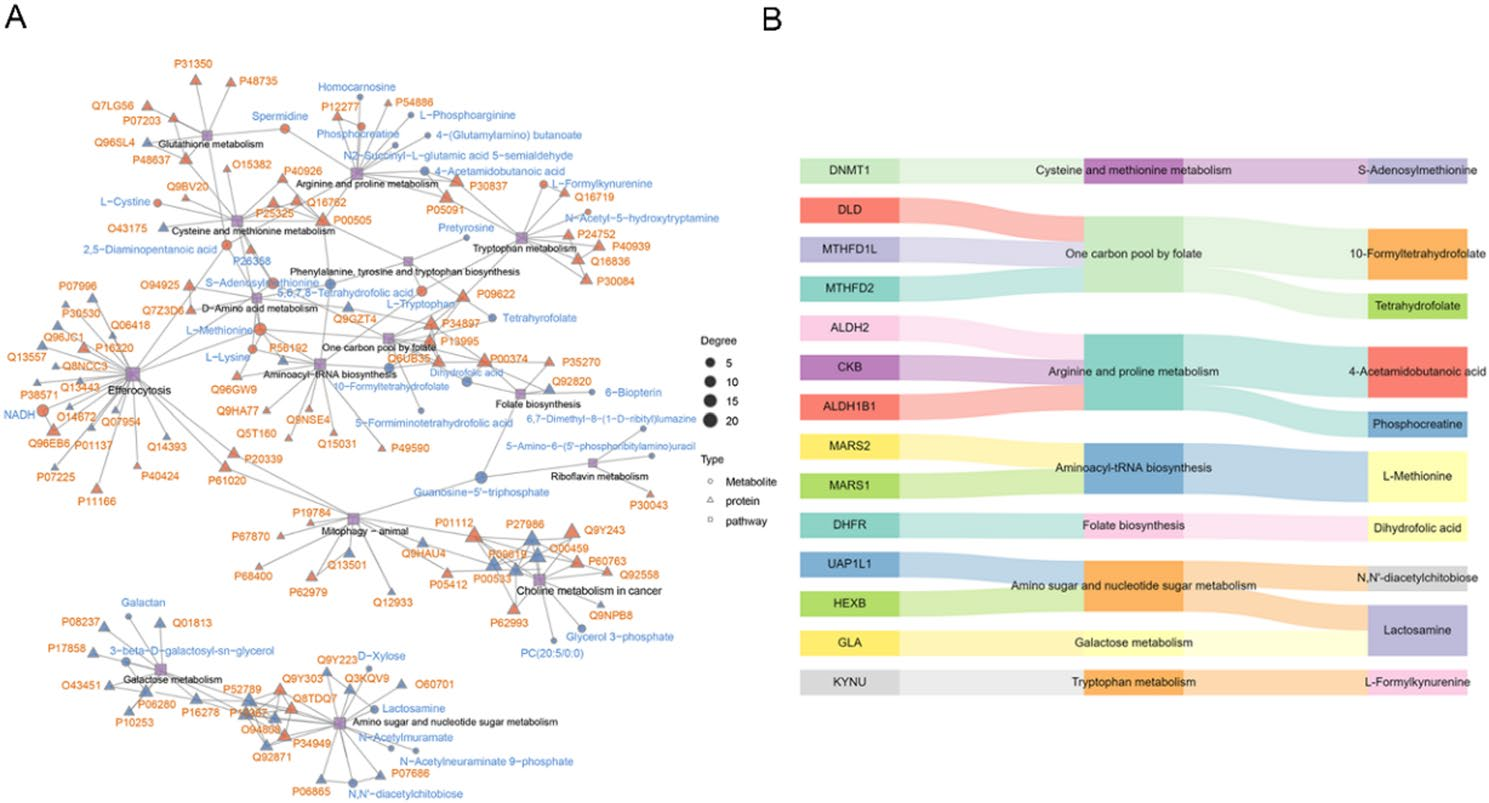
Integrative analysis of protein-metabolite-pathway associations in OGD-treated hCMEC/D3 cells. **A** Protein-pathway-metabolite interaction network. This network visualization integrates differentially expressed proteins (DEPs), differentially expressed metabolites (DEMs), and significantly enriched pathways based on Kyoto Encyclopedia of Genes and Genomes (KEGG) annotation (*q* < 0.05). Nodes: Triangles represent proteins (upregulated: orange, downregulated: blue), with size indicating node degree, and names in orange font. Circles represent metabolites (upregulated: orange, downregulated: blue), names in blue font. Diamonds represent KEGG pathways. Edges: Edges indicate known interactions among proteins, metabolites, and pathways. A total of 15 significantly enriched pathways shared by both proteomics and metabolomics are presented. **B** Sankey diagram illustrating 14 functionally linked protein-metabolite pairs. The Sankey plot presents synergistic relationships between selected proteins and metabolites through their associated KEGG pathways

To systematically resolve the potential biological associations between proteins and metabolites specifically released by hCMEC/D3 cells under OGD stress, we performed an integrative proteomic-metabolomic analysis based on the hCMEC/D3-specific DEPs (*n* = 1165) and DEMs (*n* = 2123), identified by excluding those shared with HUVECs. Firstly, KEGG pathway enrichment analysis followed by intersection analysis revealed 15 pathways commonly enriched in both proteomic and metabolomic datasets (Fig. S3). Further, based on KEGG pathway mapping, a protein-pathway-metabolite network was constructed (Fig. 3A). From this network, biologically associated protein–metabolite pairs involving 14 proteins and 10 metabolites were identified (Fig. 3B).

### Screening and validation of serological biomarkers for hypoxic response

To uncover potential serological biomarkers that are highly correlated with the hypoxic response of brain microvascular endothelial cells, we implemented a systematic protein biomarker screening strategy. The selection criteria were as follows: (1) DEPs showed a statistically significant difference (*q* < 0.05) between the OGD-treated group and the control group of hCMEC/D3 cells; (2) DEPs were identified as hub proteins in the present study; (3) DEPs were annotated as secreted proteins or previously reported to be detectable in peripheral blood; and (4) for metabolism-related proteins, inclusion in biologically associated protein-metabolite pairs identified in the integrative analysis was required. Based on these criteria, a total of 10 candidate biomarkers were selected: ALDH2, ITGA5, KYNU, TFRC, CD44, COL1A2, HEXB, HSPG2, THBS4, and DLD.

**Fig. 4:**
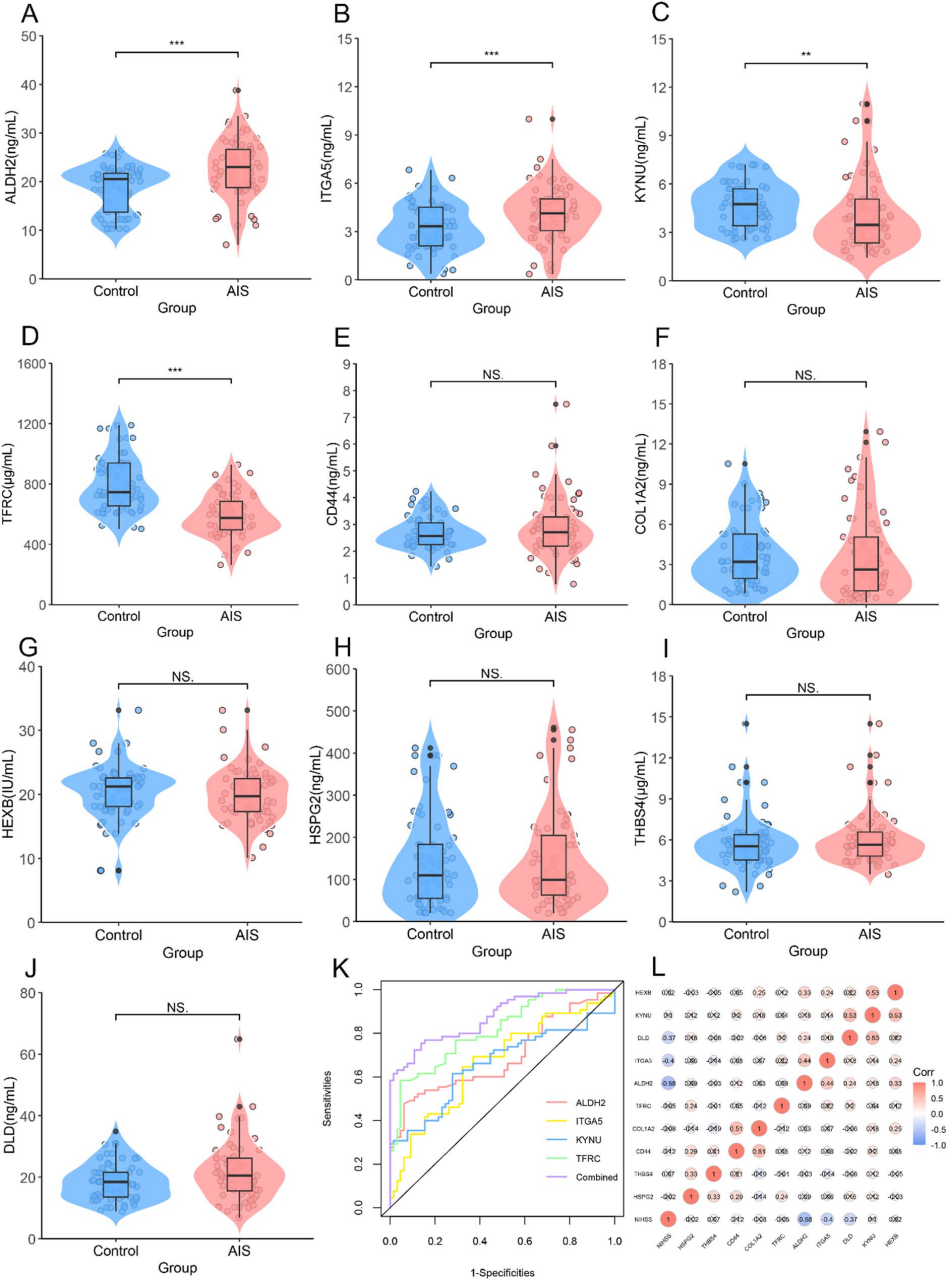
Differential expression, diagnostic utility, and NIHSS correlation of candidate biomarkers in AIS serum samples. **A**–**J** Violin plots showing the distribution of serum levels for 10 candidate biomarkers in AIS patients and healthy controls. Each plot includes boxplots (median and interquartile range) and individual data points. *P < 0.05, **P < 0.01, **P < 0.001; NS: not significant. **K** ROC curves for four significantly altered biomarkers (ALDH2, ITGA5, KYNU, TFRC) and their combination. AUC indicates diagnostic performance in distinguishing AIS from healthy controls. **L** Correlation heatmap illustrating the associations between the candidate biomarkers and baseline neurological deficit, measured by the National Institutes of Health Stroke Scale (NIHSS). Color gradient represents correlation coefficients (red: positive correlation; blue: negative correlation)

Subsequently, we performed serological validation of these 10 candidate biomarkers in AIS patients and healthy controls. The baseline characteristics of the enrolled population are summarized in Table 2. The quantitative assay results showed that ALDH2, ITGA5, KYNU, and TFRC exhibited significant differences between the two groups (*P* < 0.05) (Table 2; Fig. 4A–J. ROC analysis was performed to evaluate the diagnostic potential of the four candidate biomarkers (ALDH2, ITGA5, KYNU, and TFRC). The results showed that TFRC had the highest AUC value of 0.816 (95% CI: 0.744–0.877). A combined four-biomarker model achieved an AUC of 0.876 (95% CI: 0.818–0.934) (Fig. 4K and Table S2).

**Table 2:** Baseline characteristics and expression of 10 candidate biomarkers in AIS patients and healthy controls

Variables	Total (*n* = 130)	Con (*n* = 65)	AIS (*n* = 65)	*P*-value
Age	62 (58, 71)	60 (58, 67)	66 (58, 74)	0.07
gender, n (%)				0.111
Female	56 (43)	33 (51)	23 (35)	
Male	74 (57)	32 (49)	42 (65)	
BMI	24.08 ± 3.32	23.49 ± 3.25	24.67 ± 3.32	0.043
AF, n (%)				0.058
No	125 (96)	65 (100)	60 (92)	
Yes	5 (4)	0 (0)	5 (8)	
HTN, n (%)				< 0.001
No	78 (60)	55 (85)	23 (35)	
Yes	52 (40)	10 (15)	42 (65)	
CHD, n (%)				1
No	124 (95)	62 (95)	62 (95)	
Yes	6 (5)	3 (5)	3 (5)	
DM, n (%)				0.742
No	120 (92)	61 (94)	59 (91)	
Yes	10 (8)	4 (6)	6 (9)	
Smoking, n (%)				0.62
No	111 (85)	57 (88)	54 (83)	
Yes	19 (15)	8 (12)	11 (17)	
Drinking, n (%)				0.68
No	124 (95)	61 (94)	63 (97)	
Yes	6 (5)	4 (6)	2 (3)	
ALDH2 (ng/mL)	21.1 (18.19, 23.85)	20.51 (13.74, 21.73)	23 (18.77, 26.64)	< 0.001*
ITGA5 (ng/mL)	3.70 ± 1.64	3.23 ± 1.43	4.17 ± 1.72	< 0.001*
KYNU (ng/mL)	3.98 (3.09, 5.67)	4.75 (3.41, 5.7)	3.46 (2.34, 5.06)	0.002*
TFRC (µg/mL)	660.61 (549.57, 787.25)	745.32 (654.52, 939.65)	575.31 (496, 728.01)	< 0.001*
CD44 (ng/mL)	2.68 (2.24, 3.17)	2.57 (2.25, 3.06)	2.71 (2.18, 3.28)	0.697
COL1A2 (ng/mL)	2.91 (1.47, 5.22)	3.2 (1.95, 5.28)	2.62 (1.04, 5.06)	0.177
HEXB (IU/mL)	20.75 (17.48, 22.46)	21.23 (18.09, 22.56)	19.71 (17.31, 22.46)	0.281
HSPG2 (ng/mL)	104.57 (57.25, 194.86)	109.51 (54.65, 183.35)	98.95 (62.95, 204.54)	0.981
THBS4 (µg/mL)	5.58 (4.59, 6.42)	5.53 (4.52, 6.38)	5.64 (4.81, 6.57)	0.43
DLD (ng/mL)	18.95 (13.93, 24.2)	18.44 (13.49, 21.57)	20.51 (15.54, 26.16)	0.07

Abbreviations: BMI, Body Mass Index; AF, Atrial Fibrillation; HTN, Hypertension; CHD, Coronary Heart Disease; DM, Diabetes Mellitus; ALDH2, Aldehyde Dehydrogenase 2; ITGA5, Integrin Subunit Alpha 5; KYNU, Kynureninase; TFRC, Transferrin Receptor; CD44, Cluster of Differentiation 44; COL1A2, Collagen Type I Alpha 2 Chain; HEXB, Hexosaminidase B; HSPG2, Heparan Sulfate Proteoglycan 2; THBS4, Thrombospondin 4; DLD, Dihydrolipoamide Dehydrogenase; Con, healthy control; AIS, acute ischemic stroke. *P < 0.05 is considered statistically significant.

### Association analysis of candidate biomarkers with AIS severity and functional prognosis

**Fig. 5:**
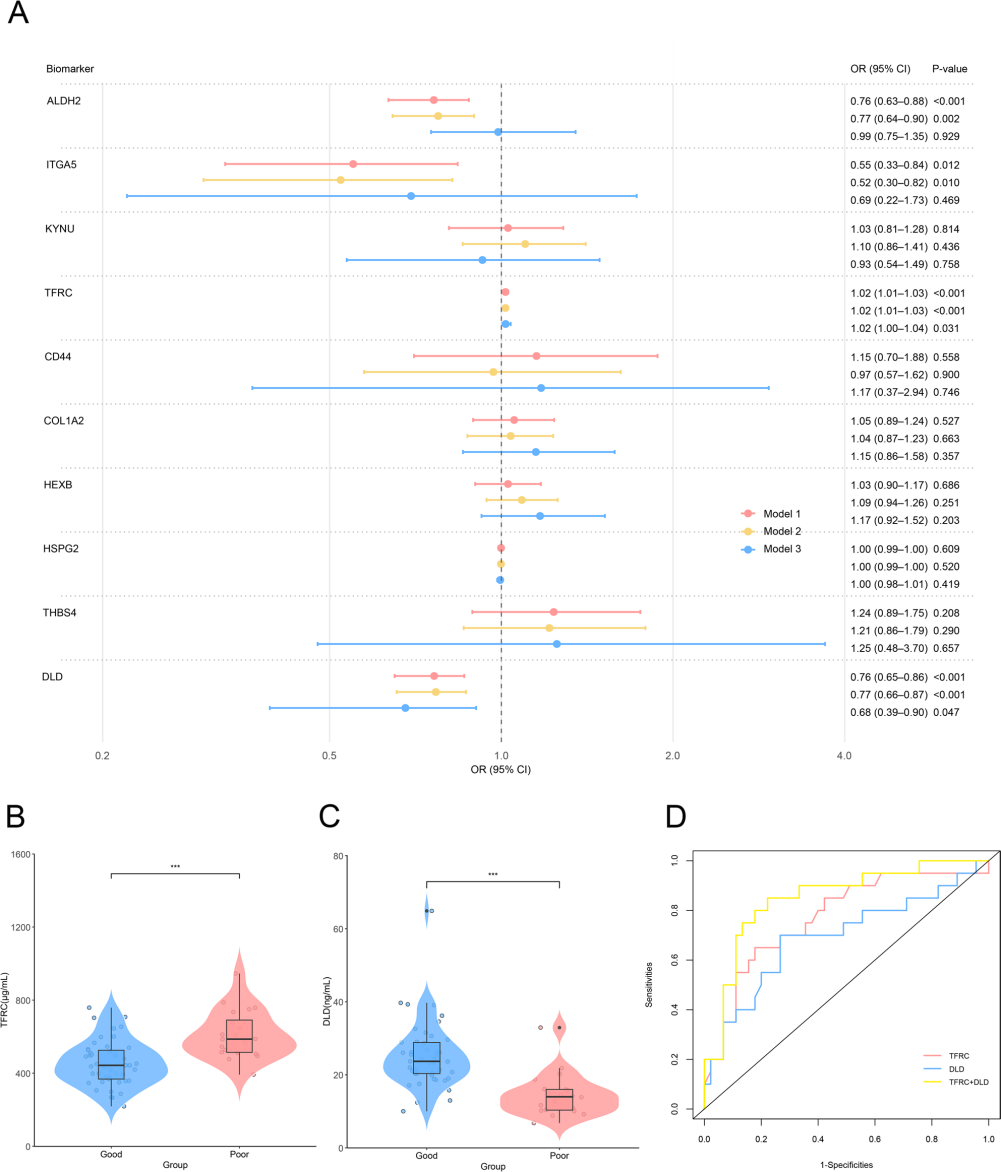
Prognostic performance of candidate biomarkers for 90-day functional outcomes in AIS patients. **A** Forest plot of logistic regression analysis assessing the association of 10 candidate biomarkers with the 90-day mRS score. The horizontal axis represents the odds ratio (OR) with 95% confidence intervals (95% CI). Biomarkers are listed on the vertical axis. Model 1 (Red): Unadjusted logistic regression model; Model 2 (Yellow): Logistic regression model adjusted for age and gender; Model 3 (Blue): Logistic regression model adjusted for age, gender, body mass index (BMI), atrial fibrillation, hypertension, coronary heart disease, diabetes mellitus, and baseline National Institutes of Health Stroke Scale (NIHSS) score. *P* < 0.05 was considered statistically significant. **B**–**C** Violin plots illustrate the differential expression of TFRC and DLD between the good-prognosis and poor-prognosis groups. The blue violin represents the good prognosis group, and the red violin represents the poor prognosis group. The central box plot indicates the median and interquartile range of biomarker expression levels. Statistical significance was assessed using the Mann-Whitney U test. ***P < 0.001. **D** ROC curves for TFRC and DLD, as well as their combination, in predicting 90-day functional outcomes. The AUC represents the discriminatory ability of the biomarkers, with a higher AUC indicating better prognostic predictive performance

Furthermore, to explore the clinical relevance of the 10 candidate biomarkers, we assessed their associations with neurological deficit severity at admission and functional outcomes at 90 days in patients with AIS. Firstly, Spearman correlation analysis revealed that ALDH2, ITGA5, and DLD were negatively correlated with baseline NIHSS scores (*r* = −0.58, −0.40, −0.37, *P* < 0.05) (Fig. 4L). Next, between good (mRS ≤ 2) and poor (mRS > 2) prognosis groups, our analysis revealed significantly downregulated expression of ALDH2, ITGA5, and DLD, whereas TFRC showed increased expression in the poor prognosis subgroup (Table 3). Univariate logistic regression analysis showed that all four of these markers were associated with poor 90-day functional prognosis (*P* < 0.05). These correlations remained significant after adjusting for age and gender in multivariate logistic regression analysis. Further adjusting for body mass index (BMI), hypertension, coronary heart disease, atrial fibrillation, diabetes mellitus, smoking, alcohol consumption and baseline NIHSS score, TFRC (OR = 1.02, 95% CI: 1.00–1.04, *P* = 0.031) and DLD (OR = 0.68, 95% CI 0.39–0.90, *P* = 0.047) still had independent prognostic predictive value, with TFRC as a risk factor and DLD as a protective factor (Fig. 5A). Focusing on TFRC and DLD (Fig. 5B–C), we performed ROC curve analysis to evaluate their prognostic predictive performance. The AUC of TFRC and DLD were 0.78 (95% CI 0.66–0.89) and 0.68 (0.55–0.79), respectively. The combination of the two improved the AUC to 0.89 (95% CI 0.67–0.97) (Fig. 5D and Table S3).

**Table 3:** Baseline characteristics and expression of 10 candidate biomarkers in AIS patients stratified by prognosis

Variables	Total (*n* = 65)	Good (*n* = 45)	Poor (*n* = 20)	*P*-value
Age	66 (58, 74)	64 (57, 71)	69 (60, 77)	0.09
Gender, n (%)				0.054
Female	23 (35)	12 (27)	11 (55)	
Male	42 (65)	33 (73)	9 (45)	
BMI	24.67 ± 3.32	24.31 ± 3.44	25.48 ± 2.92	0.167
AF, n (%)				0.165
No	60 (92)	43 (96)	17 (85)	
Yes	5 (8)	2 (4)	3 (15)	
HTN, n (%)				0.746
No	23 (35)	17 (38)	6 (30)	
Yes	42 (65)	28 (62)	14 (70)	
CHD, n (%)				0.222
No	62 (95)	44 (98)	18 (90)	
Yes	3 (5)	1 (2)	2 (10)	
DM, n (%)				0.361
No	59 (91)	42 (93)	17 (85)	
Yes	6 (9)	3 (7)	3 (15)	
Smoking, n (%)				1
No	54 (83)	35 (81)	17 (85)	
Yes	11 (17)	8 (19)	3 (15)	
Drinking, n (%)				0.538
No	63 (97)	42 (98)	19 (95)	
Yes	2 (3)	1 (2)	1 (5)	
NIHSS	5 (3, 15)	4 (2, 5)	16 (14, 18)	< 0.001*
ALDH2 (ng/mL)	23 (18.77, 26.64)	23.39 (18.53, 27.25)	17.93 (13.73, 22.13)	< 0.001*
ITGA5 (ng/mL)	4.17 ± 1.72	4.76 ± 1.64	2.85 ± 1.02	< 0.001*
KYNU (ng/mL)	3.46 (2.34, 5.06)	3.36 (2.34, 4.76)	3.95 (2.48, 5.10)	0.584
TFRC (µg/mL)	575.31 (496, 728.01)	485 (401.39, 568.61)	692.72 (562.07, 838.37)	< 0.001*
CD44 (ng/mL)	2.71 (2.18, 3.28)	2.62 (2.23, 2.94)	2.93 (2.17, 3.67)	0.374
COL1A2 (ng/mL)	2.62 (1.04, 5.06)	2.41 (0.85, 5.06)	3.02 (1.92, 4.98)	0.386
HEXB (IU/mL)	19.71 (17.31, 22.46)	19.39 ( 16.55, 22.03)	20.23 (17.51, 22.95)	0.677
HSPG2 (ng/mL)	98.95 (62.95, 204.54)	97.12 (59, 220.89)	105.43 (70.03, 148.40)	0.782
THBS4 (µg/mL)	5.64 (4.81, 6.57)	5.54 (4.57, 6.38)	5.83 (4.99, 6.88)	0.394
DLD (ng/mL)	20.51 (15.54, 26.16)	23.7 (20.35, 28.86)	13.99 (10.34, 16.00)	< 0.001*

BMI, Body Mass Index; AF, Atrial Fibrillation; HTN, Hypertension; CHD, Coronary Heart Disease; DM, Diabetes Mellitus; ALDH2, Aldehyde Dehydrogenase 2; ITGA5, Integrin Subunit Alpha 5; KYNU, Kynureninase; TFRC, Transferrin Receptor; CD44, Cluster of Differentiation 44; COL1A2, Collagen Type I Alpha 2 Chain; HEXB, Hexosaminidase B; HSPG2, Heparan Sulfate Proteoglycan 2; THBS4, Thrombospondin 4; DLD, Dihydrolipoamide Dehydrogenase; Good, good prognosis group; Poor, poor prognosis group. *P < 0.05 is considered statistically significant.

### Expression and secretion profiles of TFRC in hCMEC/D3 under OGD conditions

**Fig. 6:**
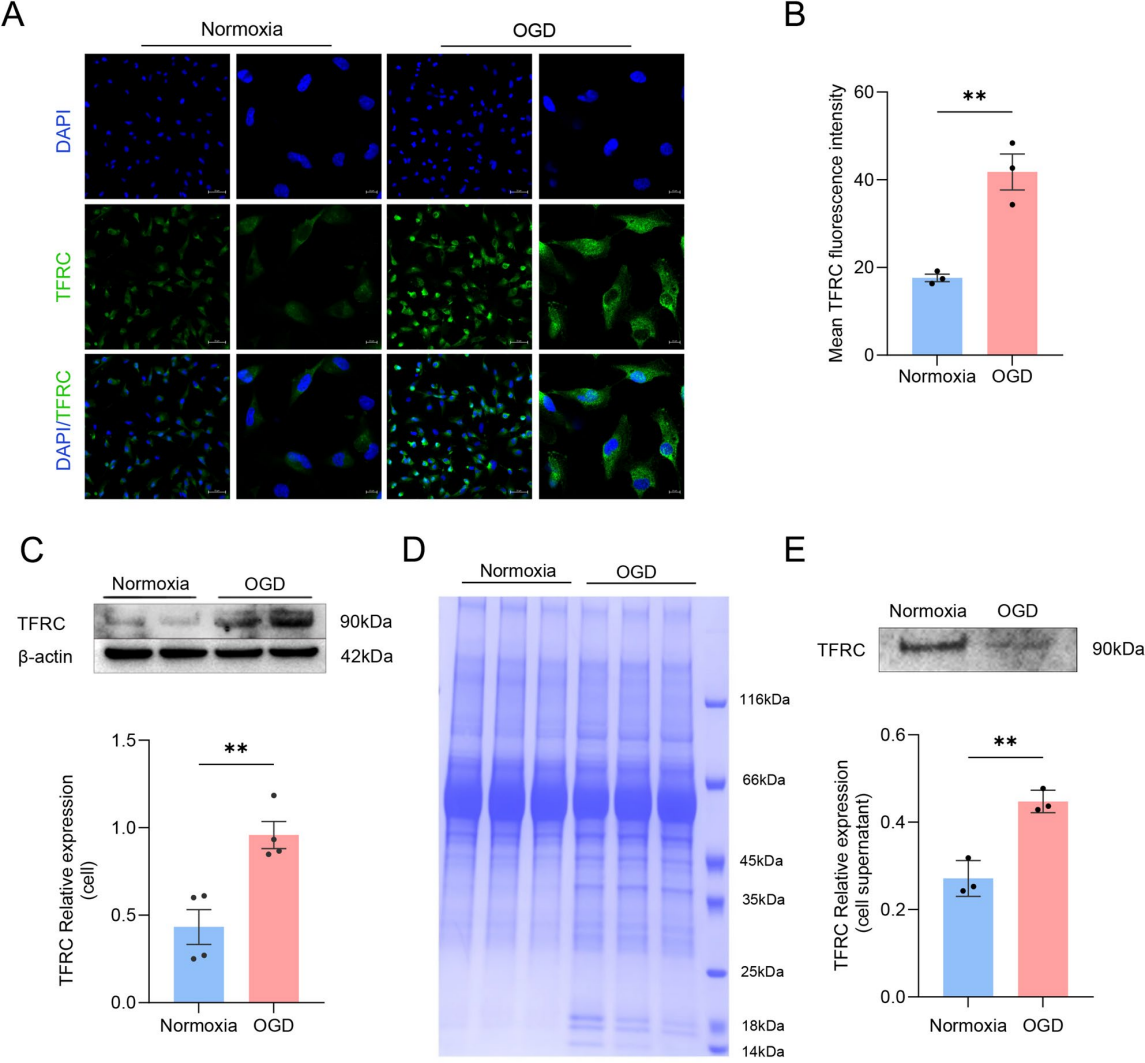
Altered TFRC expression and secretion in OGD-treated hCMEC/D3 cells. **A** Representative confocal immunofluorescence images of hCMEC/D3 cells stained for TFRC (FITC, green) and nuclei (DAPI, blue) under normoxic and oxygen-glucose deprivation (OGD) conditions. Images were acquired at both 20× (scale bar: 50 μm) and 63× oil immersion (scale bar: 10 μm) magnifications. **B** Quantification of TFRC mean fluorescence intensity under normoxia and OGD. **C** Western blot and quantification analysis of TFRC expression in cell lysates under normoxic and OGD conditions. **D** SDS-PAGE with Coomassie Brilliant Blue staining showing total protein profiles in culture supernatants. **E** Western blot analysis of TFRC levels in culture supernatants, with quantification normalized to total protein loading. **P < 0.01

Following the promising clinical validation of TFRC as a diagnostic and prognostic biomarker, we further investigated its expression and secretion dynamics in hCMEC/D3 cells under OGD conditions. Confocal immunofluorescence imaging showed that TFRC was predominantly localized at the plasma membrane and cytoplasm in both normoxic and OGD-treated cells. Quantitative analysis of fluorescence intensity indicated that TFRC expression was significantly increased in the OGD group compared to normoxia (Fig. 6A, B). Consistently, Western blot analysis revealed a significant upregulation of TFRC protein in cell lysates after OGD treatment (Fig. 6C). In contrast, TFRC levels in the culture supernatant were decreased under OGD conditions relative to normoxia (Fig. 6D, E).

## Discussion

Using LC-MS/MS, this study systematically characterized early alterations in the secretome of hCMEC/D3 cells under short-term OGD. By comparing these profiles with those of non-brain-derived HUVECs, we identified hCMEC/D3-specific secreted proteins and metabolites in response. Functional enrichment analysis revealed that these molecules were primarily involved in metabolic reprogramming, antioxidant defense, iron homeostasis, and ECM remodeling, suggesting that brain microvascular endothelial cells may engage multilayered adaptive pathways to maintain BBB integrity during early hypoxic stress. Further integrated proteomic and metabolomic analysis identified 14 protein-metabolite pairs with potential functional synergistic relationships. Importantly, through a multi-criteria screening strategy, 10 candidate markers with potential for serum-based applications were selected, including ALDH2, ITGA5, KYNU, TFRC, CD44, COL1A2, HEXB, HSPG2, THBS4, and DLD. Preliminary validation in serum samples from patients with AIS and healthy controls showed that ALDH2, ITGA5, KYNU, and TFRC were significantly differentially expressed between groups, with TFRC demonstrating promising diagnostic performance alone or in combination with the other three markers. Further multivariate logistic regression analysis indicated that elevated TFRC was independently associated with poor 90-day outcomes, whereas higher DLD levels correlated with a good prognosis.

Our integrated analysis revealed that early hypoxic stress induces coordinated metabolic and antioxidant adaptation in hCMEC/D3 cells, potentially supporting BBB homeostasis during ischemia. In particular, OGD-treated hCMEC/D3 cells exhibited enhanced TCA cycle activity, accompanied by upregulated degradation of branched-chain amino acids (valine, leucine, and isoleucine) and increased fatty acid β-oxidation. These metabolic changes may reflect an adaptive reprogramming strategy aimed at sustaining ATP synthesis under hypoxic conditions [24, 25], which are critical for maintaining cell homeostasis during the early ischemic stage. Additionally, the tryptophan metabolism pathway was significantly enriched. Its downstream metabolites may activate the aryl hydrocarbon receptor (AhR) signaling axis, thereby modulating inflammatory responses and oxidative stress to help maintain NVU homeostasis [26–28]. Concordantly, the upregulation of cysteine and methionine metabolism, together with increased glutathione biosynthesis capacity, further supports an enhanced redox buffering potential under OGD conditions [29]. Importantly, among the differentially secreted proteins, DLD, ALDH2, and KYNU emerged as key regulators of mitochondrial metabolism and oxidative stress responses. Their extracellular presence under OGD conditions highlights not only their potential as early circulating biomarkers but also as mechanistically relevant indicators of hypoxia-induced metabolic remodeling.

DLD serves as the common E3 subunit of multiple mitochondrial dehydrogenase complexes, including pyruvate dehydrogenase, α-ketoglutarate dehydrogenase, and branched-chain α-keto acid dehydrogenase complexes [30, 31]. Through its central role in mitochondrial metabolism, DLD supports both cellular energy production and maintains NAD⁺/NADH redox balance [32], which are essential for cell homeostasis. Given the high metabolic demands of brain endothelial cells and their reliance on mitochondrial function, DLD likely functions as a central regulatory node in the adaptive response of the HBMECs to ischemic stress [33]. Although DLD was robustly secreted under OGD conditions in vitro, its serum levels were not significantly elevated in AIS patients within the first 24 h of onset. This discrepancy may reflect differences in the timing or mechanism of DLD release in vivo, as it is primarily a mitochondrial matrix protein, and its appearance in circulation may be delayed, transient, or restricted by the intact BBB. Nevertheless, its expression was associated with better 90-day functional outcomes. These findings support the notion that DLD may represent an early protective metabolic enzyme that contributes to BBB homeostasis and neurological recovery by preserving mitochondrial energy metabolism in response to cerebral ischemia. Further studies are needed to elucidate the precise mechanisms and temporal dynamics of DLD release in ischemic stroke, which may help optimize its clinical utility as a circulating biomarker.

ALDH2 is a key mitochondrial dehydrogenase with increasing evidence supporting its protective function in cerebrovascular disease. It maintains mitochondrial function and endothelial homeostasis by removing toxic endogenous aldehydes, such as 4-hydroxy-2-nonenal (4-HNE), thereby mitigating lipid peroxidation-induced cellular damage [34]. It has been shown that 4-HNE is significantly elevated in the plasma of animal models and patients with IS, and exacerbates neuronal and cerebral microvascular endothelial cell damage [35]. Enhancement of ALDH2 activity effectively reduces 4-HNE accumulation, alleviates mitochondrial dysfunction, reduces infarct volume, and promotes neurological recovery [35]. Epidemiologic evidence further supports the neuroprotective effects of ALDH2. An 8-year prospective cohort study found that elevated baseline plasma 4-HNE levels are associated with an increased risk of stroke onset [36]. Consistent with these findings, our study observed significant upregulation of ALDH2 in the secretome of hCMEC/D3 cells under OGD conditions, as well as in the serum of AIS patients. These findings suggest that ALDH2 may participate in early metabolic remodeling and antioxidant responses to hypoxia in brain microvascular endothelial cells, and holds potential as an early biomarker of IS.

KYNU is a key enzyme in the tryptophan–kynurenine pathway (KP). It catalyzes the conversion of tryptophan into nicotinamide adenine dinucleotide (NAD⁺), a critical cofactor for cellular energy metabolism, redox balance, and DNA repair [37, 38]. One of KYNU’s substrates, 3-hydroxykynurenine (3-HK) [39], is capable of crossing the BBB and can be further metabolized into 3-hydroxyanthranilic acid (3-HAA) and quinolinic acid (QUIN) [40]. QUIN is known to exert neurotoxic effects by promoting reactive oxygen species (ROS) production and enhancing extracellular glutamate levels [41]. Under stress conditions, KP activation is induced in brain microvascular endothelial cells. Endothelial-derived kynurenine (KYN) can be taken up by neighboring macrophages and microglia, where it is further metabolized into neurotoxic derivatives such as QUIN [42]. Increasing evidence indicates that KP is activated in IS and is closely associated with inflammatory responses, neuronal injury, and poor outcomes [43–46]. Clinical studies showed that elevated plasma KYN/tryptophan (KYN/TRP) ratios have been observed in IS patients and correlate positively with NIHSS scores, infarct volume, and 90-day mRS scores [43]. In our study, OGD treatment led to significant upregulation of KYNU and its upstream metabolite L-formylkynurenine in the secretome of hCMEC/D3 cells, suggesting that endothelial cells may initiate early metabolic adaptations through KP activation in response to hypoxic stress. In contrast, serum KYNU levels were found to be decreased within 24 h of symptom onset in AIS patients. The observed discrepancy between in vitro and in vivo KYNU levels likely reflects systemic regulatory mechanisms beyond localized endothelial responses. AIS triggers a systemic inflammatory response driven by damage-associated molecular patterns (DAMPs) released from injured brain tissue [47, 48]. After entering the circulation, these DAMPs activate peripheral immune cells and promote the production of pro-inflammatory cytokines such as IFN-γ and TNF-α, which in turn induce indoleamine 2,3-dioxygenase 1 (IDO1) expression in dendritic cells, macrophages, and monocytes [49–51]. IDO1 is the rate-limiting enzyme in the KP, catalyzing the conversion of tryptophan to KYN [52]. The resulting increase in intracellular KYN availability may enhance downstream KP metabolism through KYNU, which may partially influence circulating levels of KYNU. Although extrahepatic KP activity in the brain or immune cells may be upregulated during ischemic or inflammatory stress, hepatic KP activity also plays an important role in systemic kynurenine metabolism [53, 54]. Over 90% of KP activity occurs in the liver, which governs systemic tryptophan metabolism and serves as the primary site for the clearance of KP intermediates, including KYNU [54]. Importantly, as kynurenine and its metabolites can cross the BBB, the central KP does not function in isolation. Furthermore, both branches of the KP are regulated by cytokines and intercellular signaling, which highlights the complex interplay between systemic and central kynurenine metabolism [38]. Therefore, as a downstream KP enzyme, circulating KYNU levels may be influenced by immune-mediated regulation, hepatic metabolism, and central KP activity, which together underscore the need to interpret circulating KYNU within the context of systemic KP dynamics.

ECM remodeling and reduced cell adhesion capacity are considered key mechanisms underlying vascular barrier dysfunction following hypoxic injury [55]. Our functional enrichment analysis revealed that the downregulated DEPs in hCMEC/D3 cells following OGD treatment were significantly enriched in the ECM-receptor interaction pathway, including critical structural components such as COL1A2, ITGA5, and THBS4. The reduced expression of these proteins may compromise basement membrane integrity and weaken endothelial adhesion, thereby contributing to early BBB instability. Interestingly, we also observed significant enrichment of the one-carbon pool by folate and folate biosynthesis pathways. This finding aligns with our previous findings in the plasma metabolomic profiles of AIS patients [56]. Dysregulation of folate metabolism may alter the levels of S-adenosylmethionine (SAM), thereby influencing DNA methylation status. This epigenetic modulation can regulate the expression of tight junction proteins, adhesion junction components, and angiogenic factors, all of which are critical for the BBB repair process [57].

The ITGA5 gene encodes the α5 subunit of the α5β1 integrin heterodimer, a transmembrane glycoprotein receptor that binds to ECM components and plays a key role in regulating the adhesion between cells and ECM [58]. Previous studies have highlighted that α5β1 integrin contributes significantly to the maintenance of BBB integrity, the regulation of endothelial tight junction protein expression, and the facilitation of angiogenesis following IS [59–62]. In animal models of cerebral ischemia, α5β1 integrin is significantly upregulated in cerebral endothelial cells [62]. Functional studies further demonstrate that knockdown of ITGA5 inhibits the proliferation of brain endothelial cells [61] and delays angiogenesis after stroke [62]. At the mechanistic level, α5β1 integrin interacts with the domain V (DV) fragment of the ECM proteoglycan perlecan. This binding event stimulates the release of vascular endothelial growth factor (VEGF) from brain endothelial cells, thereby promoting angiogenesis [63, 64]. In parallel, inhibition of β1 integrin downregulates key tight junction proteins such as claudin-5, which increases microvascular permeability and exacerbates BBB disruption [65]. In our study, ITGA5 expression was downregulated in the secretome of hCMEC/D3 cells following OGD treatment, whereas its levels were significantly elevated in the serum of AIS patients. This discrepancy may reflect the contrast between the localized, cellular response to short-term hypoxic stress in vitro and the systemic, multicellular reparative mechanisms activated in vivo. Notably, serum ITGA5 levels exhibited a negative correlation with baseline NIHSS scores, suggesting a potential association with less severe neurological deficits. Although its predictive value for 90-day functional outcome was attenuated after multivariable adjustment, the observed trend supports its potential as an early marker of cerebrovascular stability. These findings merit validation in larger, well-characterized cohorts.

Beyond metabolic and structural responses, we further investigated TFRC, a key regulator of iron uptake and cellular stress responses. TFRC is a transmembrane glycoprotein expressed on both the apical and basolateral sides of HBMECs [66], where it facilitates iron transport from the periphery into the brain [67] and mediates the transcytosis of macromolecules across the BBB [68]. This bidirectional function is crucial for maintaining iron homeostasis and BBB integrity. Our data revealed that TFRC is upregulated in hCMEC/D3 cells during early hypoxic stress, accompanied by reduced secretion into the extracellular space. This intracellular accumulation may suggest an adaptive modulation of iron homeostasis mechanisms during the early phase of oxygen deprivation. Hypoxia is known to trigger a range of metabolic and redox adaptations that promote cellular survival. In particular, HIF-1α has been shown to upregulate TFRC and DMT1, which enhances iron uptake in response to low oxygen conditions [69, 70]. This regulatory mechanism may help maintain mitochondrial function and antioxidant capacity during the early phase of ischemia, through a moderate and controlled increase in intracellular iron availability. However, this tightly regulated response may become detrimental if prolonged. In the later stages of ischemia, BBB disruption and dysregulated iron metabolism often lead to iron overload in the brain, which exacerbates ROS production and lipid peroxidation through the Fenton reaction, ultimately contributing to ferroptosis [71–73]. Consistent with this, experimental stroke models have shown that TFRC expression remains elevated during the reperfusion phase, further aggravating cellular injury [74, 75]. Collectively, these observations raise the possibility that TFRC may act as a time-dependent regulator linking iron metabolism to BBB dysfunction during ischemic progression. Importantly, the decreased TFRC levels observed in the OGD secretome parallel the reduced serum levels detected in AIS patients within 24 h of onset. This consistency between in vitro and clinical data suggests the translational potential of TFRC as a marker of early endothelial stress. Furthermore, our clinical data indicate that circulating TFRC shows promising diagnostic performance and is independently associated with 90-day poor functional outcomes. These findings may support its potential value as both a serum biomarker and a candidate therapeutic target in AIS.

Several candidate biomarkers, including CD44, COL1A2, HEXB, HSPG2, and THBS4, were initially identified in the discovery phase. However, these candidates failed to exhibit significant differential expression in serum samples from clinical validation. These negative findings may be attributed to a combination of biological and technical factors. First, the localization and structural properties of COL1A2, HSPG2, and THBS4 likely limit their release into circulation in the early phase of AIS. As major extracellular matrix components, they are predominantly localized within the vascular basement membrane. Given their large molecular size [76, 77], a significant elevation in serum levels generally requires substantial disruption of the BBB. However, within 24 h of AIS onset, structural BBB damage may still be limited, which may explain the lack of detectable increase in patient serum samples. In contrast, the absence of significant changes in CD44 levels may be attributed to its transient presence in circulation and limited assay sensitivity. As membrane-associated proteins, CD44 can be rapidly shed through proteolytic cleavage, resulting in soluble forms that are susceptible to degradation in vivo [78]. Additionally, during the early phase post-stroke, the circulating CD44 level may remain below the detection threshold of conventional assays, thereby limiting its detectability in peripheral blood. HEXB, a lysosomal enzyme, is primarily released under conditions of cellular stress or injury. While its increased levels in cell supernatants likely reflect enhanced lysosomal activity or endothelial damage following OGD, peripheral blood concentrations may remain unchanged due to limited release into circulation, rapid systemic clearance, or enzymatic degradation in vivo. Finally, inter-individual biological variability, particularly within a relatively small cohort, may limit the statistical power to detect subtle differences in circulating protein levels between groups. Taken together, these negative validation results highlight a broader challenge beyond our specific candidates. Molecules identified in vitro or in CNS biofluids often fail to reach detectable or clinically relevant levels in peripheral blood. Driven by BBB constraints, low circulating abundance, and assay limitations, these challenges highlight the need for ultra-sensitive detection platforms and peripheral surrogate markers to advance the clinical translation of CNS biomarkers.

Although this study offers mechanistic insights into the early endothelial adaptive responses to hypoxia and proposes a panel of secreted biomarkers with clinical potential for IS, several limitations should be acknowledged. First, the present study employed the hCMEC/D3 cell line, other than primary HBMECs, as an in vitro model. Although this cell line offers practical advantages such as reproducibility and ease of manipulation, it differs from primary HBMECs in key respects, including tight junction integrity, gene expression profiles, and responses to ischemic stress. Second, as a monoculture, the hCMEC/D3-based system cannot fully recapitulate the complex NVU environment present in vivo. The NYU includes pericytes, astrocytes, neurons, and extracellular matrix components that work together to maintain BBB function. Furthermore, the ischemic microenvironment in vivo is highly dynamic, involving changes in blood flow, oxygen and nutrient gradients, and immune responses, all of which are absent in the in vitro model. These discrepancies may contribute to differences between in vitro findings and clinical outcomes, emphasizing the need for cautious interpretation. Third, the clinical validation was based on a relatively small sample size, and an independent external cohort or stratified analysis by stroke subtypes was not included, which may limit the generalizability of our findings. Moreover, the temporal mismatch between the in vitro OGD treatment (6 h) and clinical serum sampling (within 24 h post-stroke) may affect the interpretation of dynamic biomarkers. While the 24-hour window is clinically relevant for AIS diagnosis and early evaluation, it may not fully reflect the acute or transient molecular responses captured in the in vitro model. This highlights the importance of better characterizing the temporal trajectories of candidate biomarkers. Finally, the diagnostic performance of these biomarkers was assessed only against healthy controls, and their specificity for AIS relative to other disorders featuring BBB disruption remains to be established.

To address these limitations, future studies should employ more physiologically relevant models, such as co-culture systems, brain organoids, or animal models, to validate and extend the endothelial-specific mechanisms observed. Larger, multi-center clinical cohorts with external validation and stratified analyses by stroke subtypes are also necessary to enhance the generalizability and clinical applicability of the proposed biomarkers. Furthermore, longitudinal cohorts at multiple time points, including early phases at 6 and 12 h post-stroke, are essential to characterize temporal patterns of biomarker expression and assess their prognostic value. Additionally, the ability of these biomarkers to distinguish AIS from other conditions associated with BBB dysfunction, such as intracerebral hemorrhage, multiple sclerosis, or neurodegenerative diseases, should be evaluated. We believed that these efforts would improve understanding of early BBB endothelial dysfunction in IS and facilitate the development of improved diagnostic, prognostic, and therapeutic strategies.

## Supplementary Information

Below is the link to the electronic supplementary material.

Supplementary Material 1

## Data Availability

The datasets used and analyzed during the current study are available from the corresponding author upon reasonable request. The proteomic datasets generated during the current study have been deposited in the ProteomeXchange Consortium ( [http://proteomecentral.proteomexchange.org](http:/proteomecentral.proteomexchange.org) ) via the iProX partner repository with the accession numbers PXD066395 and PXD066500.
