# Supplementary material for: Early hypoxia-induced secretome remodeling reveals adaptive mechanisms and biomarkers of blood-brain barrier dysfunction in ischemic stroke
Source: Mol Brain. 2026 Feb 16;19:19. doi: 10.1186/s13041-026-01283-5 (PMC13015154; doi:10.1186/s13041-026-01283-5)
Supplement: Supplementary file 1 — Supplementary Material 1 [file 13041_2026_1283_MOESM1_ESM.docx]

**Supplementary Material**

1. Table S1 Overview of 28 significantly enriched pathways identified from proteomics analysis in hCMEC/D3
2. Fig. S1 Protein-protein interaction (PPI) network of 222 hypoxia-responsive proteins identified in hCMEC/D3
3. Fig. S2 Multivariate analysis of metabolomic profiles in OGD-treated and control groups
4. Fig. S3 Sankey diagram illustrating shared pathway enrichment between proteome and metabolome
5. Table S2 Diagnostic performance of four differentially expressed biomarkers and their combination for AIS
6. Table S3 Prediction performance of TFRC, DLD, and their combination for 90-day functional prognosis

Table S1 Overview of 28 significantly enriched pathways identified from proteomics analysis in hCMEC/D3

| ID | Term | ListHits | ListTotal | PopHits | *P*-value | *q*-value | Score |
| --- | --- | --- | --- | --- | --- | --- | --- |
| Up |  |  |  |  |  |  |  |
| hsa03010 | Ribosome | 47 | 382 | 137 | 1.16E-29 | 3.40E-27 | 7.61 |
| hsa00280 | Valine, leucine and isoleucine degradation | 20 | 382 | 48 | 3.85E-15 | 5.64E-13 | 9.24 |
| hsa05171 | Coronavirus disease - COVID-19 | 47 | 382 | 295 | 1.47E-14 | 1.43E-12 | 3.53 |
| hsa00020 | Citrate cycle (TCA cycle) | 12 | 382 | 31 | 3.84E-09 | 2.82E-07 | 8.59 |
| hsa00071 | Fatty acid degradation | 13 | 382 | 43 | 2.76E-08 | 1.62E-06 | 6.71 |
| hsa00630 | Glyoxylate and dicarboxylate metabolism | 8 | 382 | 30 | 3.87E-05 | 1.63E-03 | 5.92 |
| hsa00620 | Pyruvate metabolism | 10 | 382 | 48 | 4.33E-05 | 1.63E-03 | 4.62 |
| hsa04144 | Endocytosis | 27 | 382 | 261 | 4.44E-05 | 1.63E-03 | 2.30 |
| hsa00640 | Propanoate metabolism | 8 | 382 | 32 | 6.42E-05 | 2.09E-03 | 5.55 |
| hsa03040 | Spliceosome | 17 | 382 | 131 | 7.65E-05 | 2.24E-03 | 2.88 |
| hsa00650 | Butanoate metabolism | 7 | 382 | 27 | 1.45E-04 | 3.86E-03 | 5.75 |
| hsa00380 | Tryptophan metabolism | 8 | 382 | 42 | 4.85E-04 | 1.18E-02 | 4.23 |
| hsa00062 | Fatty acid elongation | 6 | 382 | 28 | 1.30E-03 | 2.94E-02 | 4.75 |
| hsa00270 | Cysteine and methionine metabolism | 8 | 382 | 51 | 1.84E-03 | 3.84E-02 | 3.48 |
| hsa00410 | beta-Alanine metabolism | 6 | 382 | 31 | 2.27E-03 | 4.44E-02 | 4.29 |
| Down |  |  |  |  |  |  |  |
| hsa04142 | Lysosome | 20 | 243 | 135 | 1.30E-09 | 3.53E-07 | 5.17 |
| hsa00520 | Amino sugar and nucleotide sugar metabolism | 10 | 243 | 49 | 9.59E-07 | 1.30E-04 | 7.12 |
| hsa00052 | Galactose metabolism | 8 | 243 | 32 | 2.36E-06 | 2.10E-04 | 8.72 |
| hsa03013 | Nucleocytoplasmic transport | 14 | 243 | 111 | 3.09E-06 | 2.10E-04 | 4.40 |
| hsa00531 | Glycosaminoglycan degradation | 6 | 243 | 20 | 1.44E-05 | 7.86E-04 | 10.46 |
| hsa00051 | Fructose and mannose metabolism | 7 | 243 | 35 | 4.95E-05 | 2.24E-03 | 6.98 |
| hsa00511 | Other glycan degradation | 5 | 243 | 18 | 1.17E-04 | 4.56E-03 | 9.69 |
| hsa05205 | Proteoglycans in cancer | 15 | 243 | 205 | 7.93E-04 | 2.39E-02 | 2.55 |
| hsa00603 | Glycosphingolipid biosynthesis - globo and isoglobo series | 4 | 243 | 16 | 9.14E-04 | 2.39E-02 | 8.72 |
| hsa05014 | Amyotrophic lateral sclerosis | 22 | 243 | 371 | 9.53E-04 | 2.39E-02 | 2.07 |
| hsa05230 | Central carbon metabolism in cancer | 8 | 243 | 72 | 9.99E-04 | 2.39E-02 | 3.88 |
| hsa04066 | HIF-1 signaling pathway | 10 | 243 | 109 | 1.10E-03 | 2.39E-02 | 3.20 |
| hsa04512 | ECM-receptor interaction | 9 | 243 | 91 | 1.14E-03 | 2.39E-02 | 3.45 |


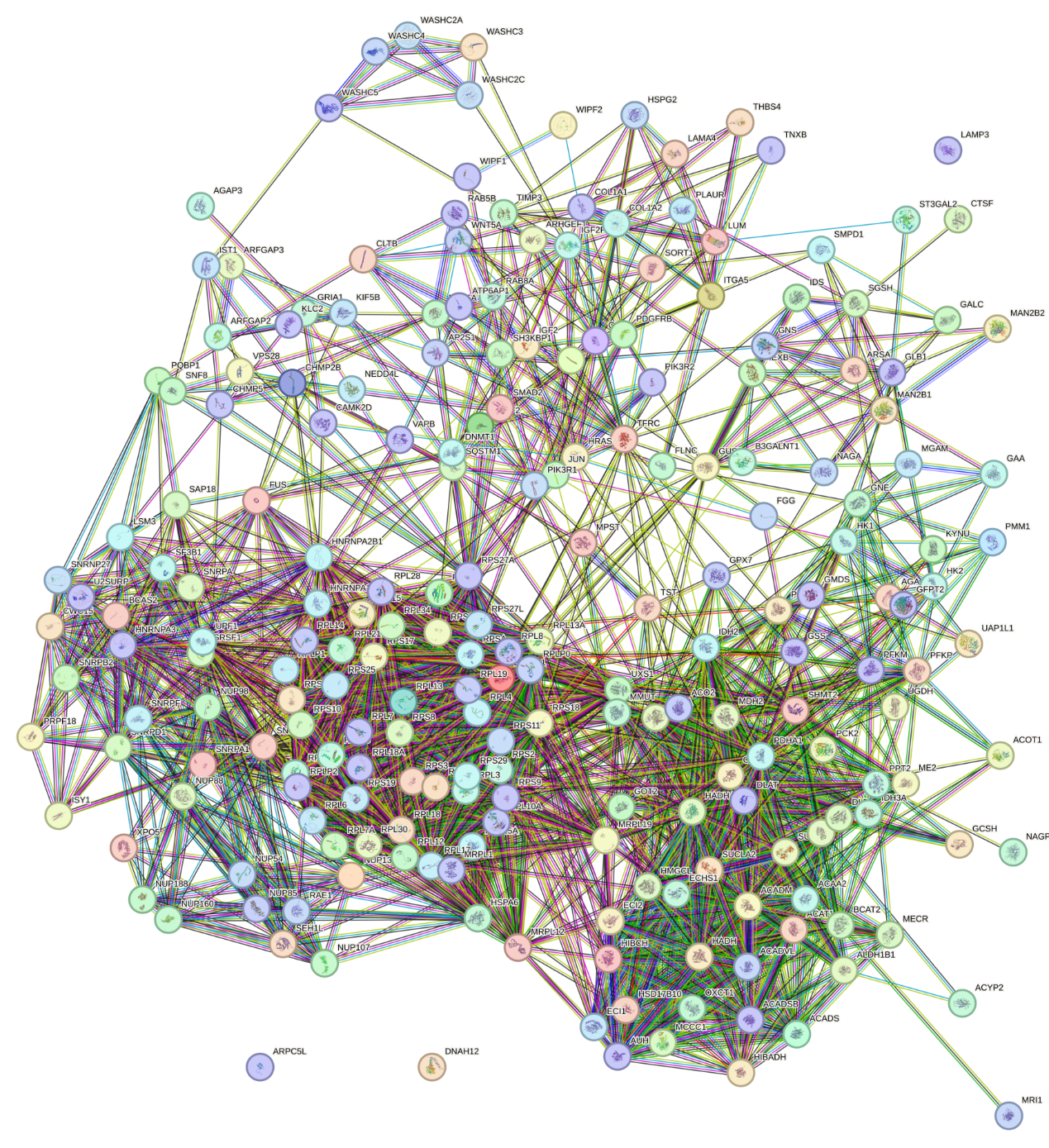


Fig. S1 Protein–protein interaction (PPI) network of 224 hypoxia-responsive proteins identified in hCMEC/D3

This network illustrates the interactions among 224 differentially expressed proteins (DEPs) enriched in 28 significant KEGG pathways (*q* < 0.05) following oxygen-glucose deprivation (OGD) in hCMEC/D3 cells. The network was constructed using the STRING database (https://string-db.org/), with interaction confidence scores ≥ 0.4. Each node represents a protein, and each edge represents a predicted or known protein–protein interaction, including physical binding and functional associations.


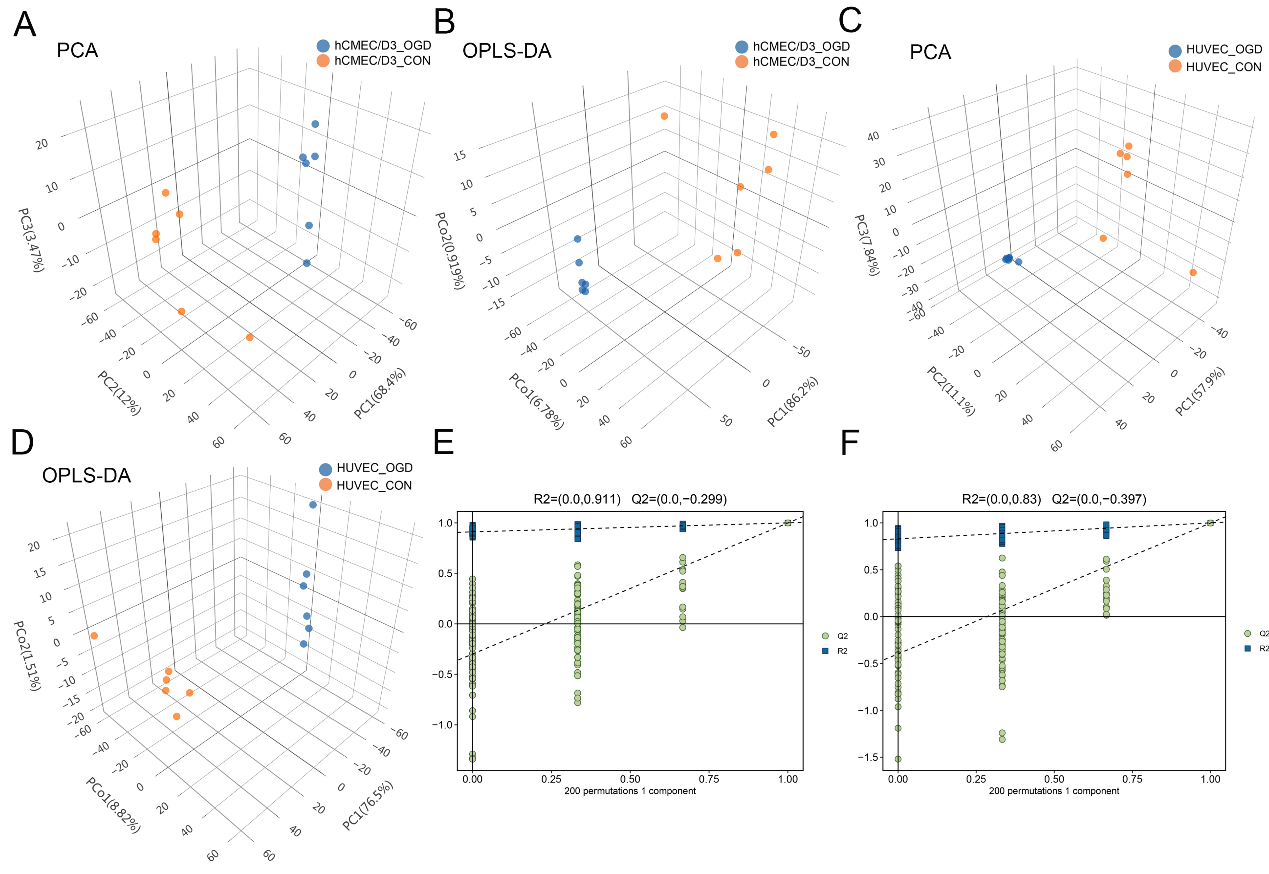


Fig. S2 Multivariate analysis of metabolomic profiles in OGD-treated and control groups

Principal Component Analysis (PCA) and Orthogonal Partial Least Squares Discriminant Analysis (OPLS-DA) score plots showing distinct separation between OGD-treated and control groups in hCMEC/D3 (A-B) and HUVEC (C-D) cells. Each point represents an individual sample, colored by group. OPLS-DA permutation test results (200 iterations) for hCMEC/D3 (E) and HUVEC (F) groups.


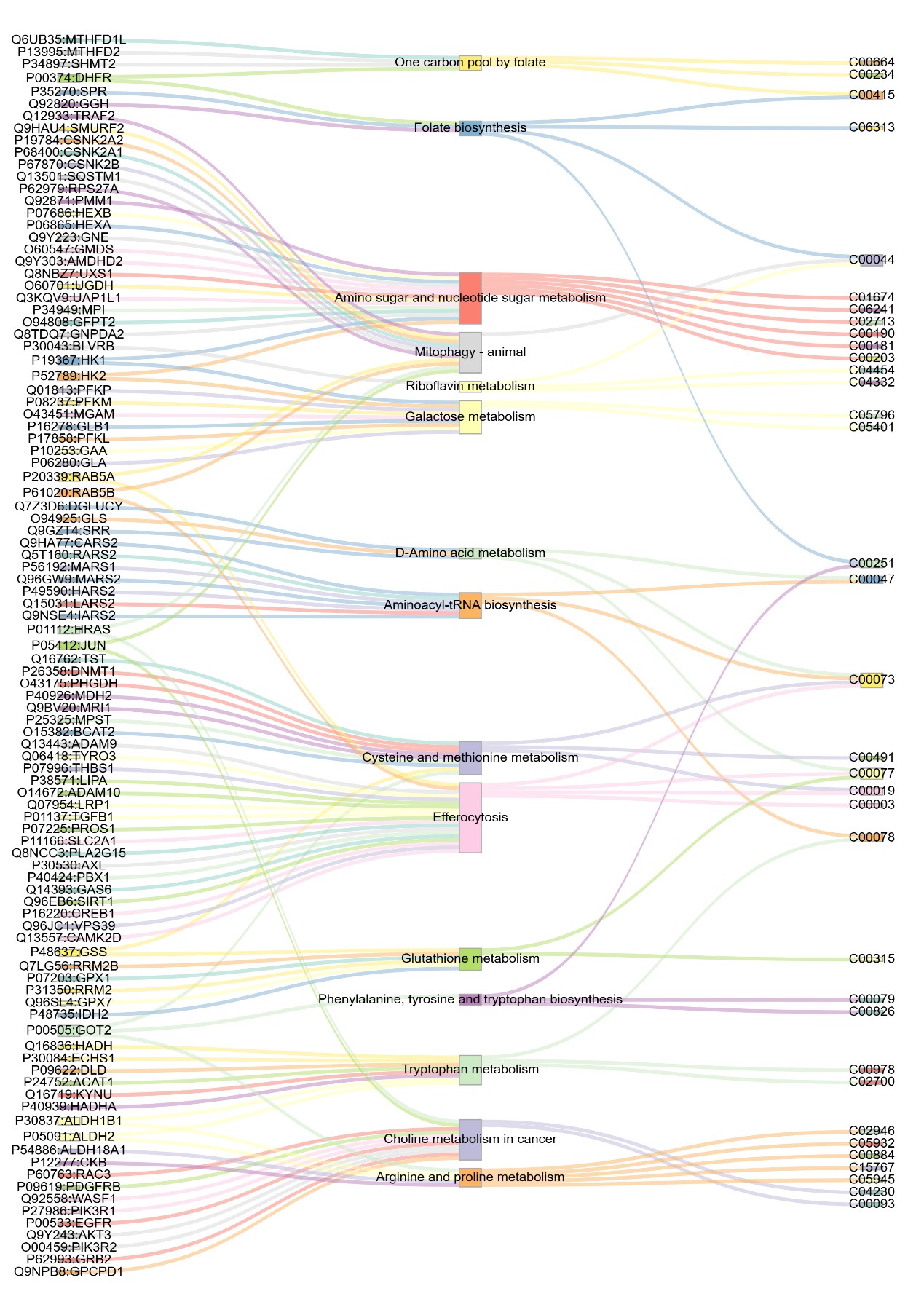
Fig. S3 Sankey diagram illustrating shared pathway enrichment between proteome and metabolome

This Sankey diagram visualizes the 15 KEGG pathways significantly enriched in both differentially expressed proteins (DEPs) and differentially expressed metabolites (DEMs). The diagram presents protein-pathway-metabolite associations within these shared pathways. On the left are DEPs, in the center are KEGG pathways, and on the right are DEMs.

Table S2 Diagnostic performance of four differentially expressed biomarkers and their combination for AIS

| Biomarker | AUC (95%CI) | You-den  index | ACC | Cut-off | SEN | SPE | NPV | PPV |
| --- | --- | --- | --- | --- | --- | --- | --- | --- |
| ALDH2 | 0.693 (0.601-0.786) | 1.42 | 0.71 | 23.36 | 0.48 | 0.94 | 0.64 | 0.89 |
| ITGA5 | 0.666 (0.572-0.760) | 1.32 | 0.66 | 3.70 | 0.65 | 0.68 | 0.66 | 0.67 |
| KYNU | 0.657 (0.561-0.754) | 1.34 | 0.67 | 3.92 | 0.62 | 0.72 | 0.65 | 0.69 |
| TFRC | 0.816 (0.744-0.877) | 1.54 | 0.77 | 600.86 | 0.58 | 0.95 | 0.70 | 0.93 |
| Combined | 0.876 (0.818-0.934) | 1.62 | 0.81 | 0.22 | 0.77 | 0.85 | 0.79 | 0.83 |

Abbreviations: AUC, area under the curve; ACC, accuracy; SEN, sensitivity; SPE, specificity;

NPV, negative predictive value; PPV, positive predictive value.

Table S3 Prediction performance of TFRC, DLD, and their combination for 90-day functional prognosis

| Proteins | AUC (95%CI) | You-den  index | ACC | Cut-off | SEN | SPE | NPV | PPV |
| --- | --- | --- | --- | --- | --- | --- | --- | --- |
| TFRC | 0.776 (0.662-0.891) | 1.47 | 0.76 | 596.04 | 0.65 | 0.82 | 0.84 | 0.62 |
| DLD | 0.677 (0.552-0.785) | 1.41 | 0.70 | 16.14 | 0.70 | 0.71 | 0.84 | 0.52 |
| Combined | 0.887 (0.673-0.966) | 1.62 | 0.80 | -0.78 | 0.85 | 0.78 | 0.92 | 0.63 |

Abbreviations: AUC, area under the curve; ACC, accuracy; SEN, sensitivity; SPE, specificity;

NPV, negative predictive value; PPV, positive predictive value.
